# Alterations by Air Pollution in Inflammation and Metals in Pleural Effusion of Pneumonia Patients

**DOI:** 10.3390/ijerph16050705

**Published:** 2019-02-27

**Authors:** Kuan-Jen Bai, Kai-Jen Chuang, Jen-Kun Chen, Cheng-Yu Tsai, You-Lan Yang, Chih-Cheng Chang, Tzu-Tao Chen, Chun-Nin Lee, Po-Hao Feng, Kuan-Yuan Chen, Kang-Yun Lee, Chein-Ling Su, Shu-Chuan Ho, Sheng-Ming Wu, Hsiao-Chi Chuang

**Affiliations:** 1 School of Respiratory Therapy, College of Medicine, Taipei Medical University, Taipei 110, Taiwan; kjbai@w.tmu.edu.tw (K.-J.B.); ylyang@tmu.edu.tw (Y.-L.Y.); leecn@shh.org.tw (C.-N.L.); 10038@s.tmu.edu.tw (C.-L.S.); shu-chuan@tmu.edu.tw (S.-C.H.); 2 Division of Pulmonary Medicine, Department of Internal Medicine, School of Medicine, College of Medicine, Taipei Medical University, Taipei 110, Taiwan; changpredictor@gmail.com (C.-C.C.); pohao.feng@gmail.com (P.-H.F.); kangyunlee68@gmail.com (K.-Y.L.); chitosanase@yahoo.com.tw (S.-M.W.); 3 Division of Pulmonary Medicine, Department of Internal Medicine, Wan Fang Hospital, Taipei Medical University, Taipei 116, Taiwan; 4 School of Public Health, College of Public Health, Taipei Medical University, Taipei 110, Taiwan; kjc@tmu.edu.tw; 5 Department of Public Health, School of Medicine, College of Medicine, Taipei Medical University, Taipei 110, Taiwan; 6 Institute of Biomedical Engineering & Nanomedicine, National Health Research Institutes, Miaoli 35053, Taiwan; jkchen@nhri.org.tw; 7 Graduate Institute of Life Sciences and School of Dentistry, National Defense Medical Center, Taipei 114, Taiwan; 8 Sleep Center, Shuang Ho Hospital, Taipei Medical University, New Taipei City 235, Taiwan; momo123163@gmail.com; 9 Division of Pulmonary Medicine, Department of Internal Medicine, Shuang Ho Hospital, Taipei Medical University, New Taipei City 235, Taiwan; 09330@s.tmu.edu.tw (T.-T.C.); a112378633@gmail.com (K.-Y.C.)

**Keywords:** air pollution, heart failure, immune function, infection, metal

## Abstract

Air pollution is known to increase the risk of pneumonia. However, the effects of air pollution on the pleural effusion of patients with pneumonia are unclear. The objective of this study was to investigate alterations in inflammatory–immune biomarkers by air pollution in patients with pneumonia by analyzing their pleural effusion. Patients who had undergone thoracentesis to drain their pleural effusion in a hospital were recruited for this study. Patients with pneumonia and those with congestive heart failure respectively served as the case and control groups. We observed that an increase of 1 ppb in one-year NO_2_ was associated with a decrease of 0.105 ng/mL in cluster of differentiation 62 (CD62) (95% confidence interval (CI) = −0.085, −0.004, *p* < 0.05) in the pleural effusion. Furthermore, we observed that an increase in one−year 1 ppb of NO_2_ was associated with a decrease of 0.026 ng/mL in molybdenum (Mo) (95% CI = −0.138, −0.020, *p* < 0.05). An increase in one-year 1 ppb of SO_2_ was associated with a decrease of 0.531 ng/mL in zinc (95% CI = −0.164, −0.006, *p* < 0.05). Also, an increase in one-year 1 ppb of O_3_ was associated with a decrease of 0.025 ng/mL in Mo (95% CI = −0.372, −0.053, *p* < 0.05). In conclusion, air pollution exposure, especially gaseous pollution, may be associated with the regulation of immune responses and changes in metal levels in the pleural effusion of pneumonia patients.

## 1. Introduction

Pneumonia is reported to be one of the leading causes of childhood deaths worldwide, accounting for about 1.3 million deaths among children under five years old annually in 2010 and 2011 [1]. Recent reports highlighted that there are many risk factors that may increase the risk of pneumonia onset. Air pollution, for example, is recognized as being one of the risk factors for pneumonia [2]. A study correlated 44,801 hospital outpatient visits for respiratory diseases with air pollution exposure in China; those results indicated that an interquartile range (IQR; 33.61 μg/m^3^) of particulate air pollution increased hospital outpatient visits (15.41%; 95% confidence interval (CI): 10.99%, 20.01%) [3]. A population-based case-control study in Canada showed that exposure to nitrogen dioxide (NO_2_) and particulate matter (PM) of <2.5 μm in aerodynamic diameter (PM_2.5_) was associated with hospitalizations of older adults for community-acquired pneumonia (OR = 2.26; 95% CI: 1.20, 4.24) [4]. Results of a meta-analysis showed an association between air pollution (including particulate and gaseous pollutants) and hospitalizations of children due to pneumonia [5]. Although ample epidemiological evidence has shown associations between air pollution and pneumonia, there is a paucity of biological evidence from patients with pneumonia confirming the association and investigating the possible underlying mechanisms. Immunosuppression was reported to be one important mechanism that occurs due to air pollution [6]. There is increasing evidence that air pollution adversely influences the lungs’ defense mechanisms. Air pollutants may be involved in immune responses, leading to immune dysregulation [7,8]. Pleural effusion is excess fluid that accumulates between the visceral and parietal pleural membranes surrounding the lungs. Excessive pleural fluid can impair breathing by limiting lung expansion during ventilation. Clinically, pleural effusion results from different diseases such as pneumonia and congestive heart failure (CHF). Pneumonia causes fluid buildup in the thin space between layers of tissues that line the lungs and pleural cavity. In patients with CHF, pleural effusion occurs as a result of increased interstitial fluid in the lungs, which is due to elevated pulmonary capillary pressure. Pleural effusions are classified as either uncomplicated or complicated. Uncomplicated pleural effusions are free of serious inflammation or infection, whereas complicated pleural effusions involve significant inflammation or infection. Pleural effusions contain proteins originating from plasma filtrate released during inflammation [9,10,11]. Therefore, proteins or metals in pleural effusions can be objectively and quantitatively measured and evaluated as biomarkers of normal or pathological biological processes and exposure to environmental factors.

Metals determined in body fluids or samples are commonly used for exposure assessments. For example, urinary metals have been linked to metal fume PM_2.5_ exposure in shipyard workers [12]. The authors observed that urinary aluminum (Al), chromium (Cr), iron (Fe), and nickel (Ni) were statistically associated with urinary neutrophil gelatinase-associated lipocalin after metal fume PM_2.5_ exposure. Residents exposed to nearby e-waste burning which emitted PM of <10 μm in an aerodynamic diameter (PM_10_) were associated with higher serum levels of Cr, Ni, copper (Cu), and zinc (Zn) [13]. Although metal levels and biomarkers determined in urine and blood have been used in environmental and occupational studies, those results can only represent systemic effects after exposure to air pollution. Our previous study showed that Zn and glucose in the pleural effusion significantly changed in smokers compared to non-smokers [14]. Therefore, pleural effusion could be a good candidate for investigating the local lung environment after air pollution exposure.

A recent study reported that seven workers exposed to 5–13 months of nanoparticles (NPs) had shortness of breath and pleural effusion [15]. Importantly, NPs were identified in pulmonary epithelial and mesothelial cells as well as the pleural effusion in those workers. Consistently, pleural effusion, pulmonary fibrosis, and granulomas occurred in Wistar rats after pulmonary exposure to polyacrylate/silica NPs [16]. These observations suggest that nanoscale PM is able to induce pulmonary injury, leading to the formation of pleural effusion. However, the effects of air pollution on alterations in biomarkers and metals in the pleural effusion of patients with pneumonia remain unclear. The objective of this study was to investigate the associations between air pollution and inflammatory and immune-related biomarkers and metals in pneumonia patients.

## 2. Materials and methods

### 2.1. Ethics

This study was approved by the Ethics Committee of the Taipei Medical University Joint Institutional Review Board (Taipei, Taiwan). Methods were carried out in accordance with the approved study protocol. All subjects received written and oral information prior to inclusion and provided informed consent. This study was registered in the Chinese Clinical Trial Registry and obtained a Clinical Trials Identifier serial number (ChiCTR-ROC-16008921).

### 2.2. Study Population

Patients who had undergone thoracentesis to drain their pleural effusion in a hospital (New Taipei City, Taiwan) between June 2013 and May 2014 were recruited for this study. Patients with pneumonia and those with CHF respectively served as case and control groups. Pneumonia-caused pleural fluid is due to local inflammation/injury in the lungs; therefore, accumulating fluid builds up in the thin space between the layers of tissues that line the lungs and pleural cavity. CHF-induced pleural fluid results from systemic responses, leading to elevated pulmonary capillary pressure followed by fluid accumulation in the pleural cavity. Therefore, pleural effusions collected from subjects with a diagnosis of CHF were used as a comparison group for lung disease, as was done in previous studies [14,17]. Inclusion criteria were: (1) having been diagnosed with pneumonia or CHF and (2) being >20 and <80 years old. Exclusion criteria were: (1) patients positive for human immunodeficiency virus and (2) having undergone an invasive thoracic procedure within the past 6 months or treatment with an antineoplastic drug. A baseline screening questionnaire was administered to assess age, sex, body mass index (BMI), smoking, alcohol consumption, and respiratory comorbidities (asthma and chronic obstructive pulmonary disease (COPD)).

### 2.3. Environmental Monitoring

Air pollution data for PM_2.5_, NO_2_, SO_2_ (sulfur dioxide), and O_3_ (ozone) linked to subjects’ residential addresses were obtained from the Taiwan Air Quality Monitoring Network, operated by the Taiwan Environmental Protection Administration (http://taqm.epa.gov.tw/taqm/tw/). Data collected from the network were previously reported [18]. Briefly, data from the nearest air monitoring station, which was within 10 km of the corresponding address, were used to represent the participant’s ambient air pollution exposure. One-year average levels of PM_2.5_, NO_2_, SO_2_, and O_3_ were collected to represent individual exposure conditions to air pollution during the study period.

### 2.4. Pleural Effusion

Pleural effusion was collected and stored according to previously established guidelines [19]. Pleural fluid was collected from each participant by echo-guided aspiration. After the procedure, the remaining effusion was collected and centrifuged for 10 min at 800× *g* and 4 °C, and the supernatant was stored at −80 °C until further analysis.

### 2.5. Pleural Biochemistry

Lactate dehydrogenase (LDH) and total protein were analyzed by a laboratory at the hospital. C-X-C motif chemokine 10 (CXCL10), interferon (IFN)-γ, cluster of differentiation 14 (CD14), and CD62 were determined using BD Cytometric Bead Array tests (CA, USA) followed by analysis with a BD LSR Fortessa™ cell analyzer (Franklin Lakes, NJ, USA). The method followed the manufacturer’s instructions.

### 2.6. Pleural Metal Concentrations

Pleural metal concentrations, including Al, Cr, Fe, cobalt (Co), Cu, Zn, arsenic (As), molybdenum (Mo), silver (Ag), tin (Sn), and lead (Pb) were determined using inductively coupled plasma mass spectrometry (ICP-MS 7500cx, Agilent Technologies, Tokyo, Japan). Briefly, samples were digested with nitric acid (Fisher Scientific, USA) in a microwave digestion system (MARS Xpress 5, CEM, Matthews, NC, USA), using 1600 W of heating power to maintain 140 °C for 20 min. Samples were diluted to a final concentration of 5% nitric acid with nitric acid and deionized (>18 MΩ) water. Standard solutions (High-Purity Standards, Charleston, SC, USA), serially diluted to 0.5 ng/mL, 1.0 ng/mL, 5.0 ng/mL, 10 ng/mL, 100 ng/mL, and 1000 ng/mL, were used to prepare calibration curves for quantification. The relative percentage difference was <10%.

### 2.7. Statistical Analysis

All of the statistical analyses in this study were performed with SPSS 15.0 software (SPSS, New York, NY, USA). A normality test was conducted to examine if the data were normally distributed. Data were transformed (log_10_) if the data were not normally distributed. A Chi-squared test or Fisher’s exact test was used for comparison between nominal variables. A student’s *t*-test was used for comparisons between continuous variables. A generalized linear model (GLM) was used to investigate associations between air pollutants and biomarkers/metals in all patients. The dependent variables, i.e., LDH, total protein, CXCL10, IFN-γ, CD14, CD62, and metals in the pleural effusion, were normalized, while the independent variables were one-year PM_2.5_, NO_2_, SO_2_, and O_3_. Age, sex, BMI, smoking, and disease category (pneumonia or CHF) were adjusted for in the model. The level of significance for all statistical analyses was set to *p* < 0.05.

## 3. Results

### 3.1. Characterization of Study Subjects and Air Pollution Exposure

Sixty-three subjects with pneumonia and 27 subjects with CHF were enrolled in the present study (Table 1). Pneumonia subjects (77 ± 12 years) were significantly older than CHF subjects (66 ± 13 years; *p* < 0.001). Approximately 69.8% of pneumonia subjects were men, whereas 48.1% of CHF subjects were men. CHF subjects had a higher body mass index (BMI; 24 kg/m^2^) than pneumonia subjects (22 kg/m^2^). There were no significant differences in numbers of current smokers or alcohol consumers between the pneumonia and CHF groups. The percentages of patients with respiratory comorbidities of asthma and COPD were similar between the two groups.

The average temperature was 23.9 ± 0.1 °C and relative humidity (RH) was 74.8% ± 3.5%, during the study period. One-year average values of PM_2.5_, NO_2_, SO_2_, and O_3_ for personal exposure among the subjects are listed in Table 1. There were no significant differences in PM_2.5_, NO_2_, SO_2_, or O_3_ between subjects with pneumonia and those with CHF.

### 3.2. Biomarkers and Metals in Pleural Effusion

The levels of biomarkers and metals in pleural effusion samples are listed in Table 2. We observed that CD14 was significantly lower in pneumonia subjects compared to CHF subjects (*p* < 0.05). There was no significant difference in LDH, total protein, CXCL10, IFN-γ, or CD62 between the groups. Additionally, we observed that Al in the pneumonia group was significantly lower than that in the CHF group (*p* < 0.01). But subjects with pneumonia had higher levels of Cr in the pleural effusion than did CHF subjects (*p* < 0.05). There were no differences in Fe, Cu, Zn, As, Mo, Sn, or Pb between the pneumonia and CHF groups.

### 3.3. Associations of Pleural Biomarkers and Metals with Air Pollution

Associations of PM_2.5_, NO_2_, SO_2_, and O_3_ with the log-transformed LDH, total protein, CXCL10, IFN-γ, CD14, and CD62 levels in pleural effusion were determined after adjusting for age, sex, BMI, smoking, and disease category (pneumonia or CHF) (Table 3). We observed that an increase of 1 ppb in NO_2_ was associated with a decrease of 0.105 ng/mL (10^−0.979^ = 0.105) in CD62 (95% CI = −0.085, −0.004, *p* < 0.05). We observed no significant association of LDH, total protein, CXCL10, IFN-γ or CD14 with air pollutants.

Table 4 shows the associations of PM_2.5_, NO_2_, SO_2_, and O_3_ with log-transformed metal concentrations of pleural effusion after adjusting for age, sex, BMI, smoking, and disease category (pneumonia or CHF). We observed that an increase in 1 ppb of NO_2_ was associated with a decrease of 0.026 ng/mL (10^−1.590^ = 0.026) in Mo (95% CI = −0.138, −0.020, *p* < 0.05), and an increase in 1 ppb of SO_2_ was associated with a decrease of 0.531 ng/mL (10^−0.275^ = 0.531) in Zn (95% CI = −0.164, −0.006, *p* < 0.05). Also, an increase in 1 ppb of O_3_ was associated with a decrease of 0.025 ng/mL (10^−1.599^ = 0.025) in Mo (95% CI = −0.372, −0.053, *p* < 0.05). We observed no significant associations between air pollutants and other metals in the pleural effusion.

## 4. Discussion

The relationship between air pollution and pneumonia is still poorly understood. In the present study, for the first time, we investigated the effects of air pollution on alterations in biomarkers and metals in the pleural effusion of patients with pneumonia. The findings of this study revealed that air pollution was associated with regulation of immune responses and metal levels of the pleural effusion of patients with pneumonia.

Pneumonia causes pleural effusion due to lung inflammation and injury, whereas CHF induces pleural effusion due to elevated pulmonary capillary pressure. Therefore, pleural effusion occurring due to pneumonia represents the local lung environment. However, pleural effusion occurring due to CHF represents the systemic environment, which served as a control for pulmonary disease [14]. The one-year average PM_2.5_ mass concentration was 26.3 μg/m^3^ in both groups, which was similar to our previous study conducted in Taipei [18]. Notably, the one-year average PM_2.5_ level was relatively higher than the respective World Health Organization (WHO) PM_2.5_ guidelines of 10 μg/m^3^ for one-year average [20]. In terms of the gaseous pollutants, levels of NO_2_, SO_2_, and O_3_ were similar to those in a previous study in Taipei [18]. All three of these gaseous pollutants were lower than the WHO air quality guidelines [20]. A previous study showed that air pollution, such as PM_2.5_, was associated with hospital admissions for pneumonia in Taipei [21]. Also, PM_2.5_, NO_2_, and SO_2_ were associated with increased hospital visits for pneumonia in children in China [22]. Although an association between air pollution and pneumonia has been observed, alterations in biomarkers and metals in pleural effusion caused by air pollution remain unclear.

To understand expressions of pleural biomarkers, lung injury, and inflammatory and immune-related cytokines of the pleural effusion were determined. We found that patients with pneumonia had lower levels of CD14 than those with CHF. CD14 is expressed on the surface of alveolar macrophages, infiltrating monocytes and neutrophils, and epithelial and endothelial cells in the lungs as immune responses [23]. Soluble CD14, as determined in the present study, enhances lipopolysaccharide-induced activation of cells with low CD14 expression. Consistently, alterations in CD14 were previously reported to be associated with air pollution exposure [24,25]. However, we observed no association between air pollution and CD14. In the present study, we observed that CD62 levels were negatively associated with one-year NO_2_ exposure. CD62 is recognized as a marker of neutrophil infiltration in the lungs [26,27]. Neutrophils are one of the essential regulators of host defense in lung disease. Pneumonia, for example, is an important cause of mortality throughout the world [28]. The initial phase of bacterial pneumonia is characterized by neutrophil-mediated inflammation [28,29], which assists in removing bacteria. Notably, we observed that NO_2_ exposure was associated with a reduction in CD62 expression in the lungs of pneumonia patients. Negative regulation by NO_2_ could be due to reactive oxygen species (ROS), leading to inhibition of neutrophil recruitment [30]. Therefore, NO_2_ may suppress the removal mechanisms of bacterial infection in pneumonia, prolonging the recovery period.

We then determined pleural metal concentrations using ICP-MS and compared them between the two groups. The levels of Al and Cr significantly differed in the pleural effusion of pneumonia patients compared to CHF patients. Higher levels of pleural metals in patients with CHF may have resulted from increased permeability, leading to metal accumulation in the pleural cavity from the systemic circulation. Next, we determined associations between air pollution and metals in the pleural effusion. Metal levels in biological samples are commonly used to identify the internal dose of environmental exposure [31,32]. However, most samples are collected from systemic fluids (e.g., blood), excretions (e.g., urine), or human tissues to represent biomarkers for pulmonary exposure. In the present study, we collected pleural effusion to represent the local response of the lungs to air pollution exposure. We observed that exposure to SO_2_ was negatively associated with Zn, whereas NO_2_ and O_3_ were negatively associated with Mo in the pleural effusion. A previous study also showed that SO_2_ was associated with urinary metals (i.e., Al and rubidium) [33]. Alterations in metals of the pleural cavity may occur due to internal metals released from cells/tissues or systemic fluid after exposure. Metals were determined in pleural effusion samples (internal metal levels); thus, alterations in metals could be attributed to various factors. We suspect that the association of gases with metal levels could be due to secondary/indirect effects. For example, NO_2_, SO_2_, and O_3_ are recognized as environmental oxidants that are able to impair pulmonary tissues and cells. The injury caused by these gaseous pollutants may increase the permeability of the lungs, leading to exchange between systemic metals and the pleural cavity. Therefore, the increased permeability in the lungs could cause accumulated pleural metals to be released to circulating systems. Thus, more information obtained from the pleural effusion for exposure assessment is required for further confirmation.

There are some limitations in this study. First, there could be exposure misclassification because data from monitoring stations near the subjects’ homes were used as exposure data, but no information was available about their work places. The sample size was relatively small. Personal measurements for exposure to air pollution were not conducted. Also, no information about the patients’ occupational exposure was obtained, which might have played an important role. As the metal levels were determined in the pleural fluid, alterations in metals may be associated with disease type. We did not examine metals contained in the PM. Therefore, it is hard to identify correlations of metals between external (PM_2.5_) and internal (pleural effusion) sources after exposure.

## 5. Conclusions

In conclusion, exposure to air pollution may be associated with regulation of the immune response and metal levels. Our findings showed that the pleural effusion could represent the microenvironment of the lungs for exposure assessments in environmental studies.

## Figures and Tables

**Table 1:** Demographic characteristics of 63 patients with pneumonia and 27 patients with congestive heart failure (CHF) during the study period.

Characteristic	Pneumonia (*n* = 63) (*n*, %)	CHF (*n* = 27) (*n*, %)	*p* Value
Age (years) ± SD ^a^	77 ± 12	66 ± 13	<0.001
Male ^b^	44 (69.8)	13 (48.1)	0.050
BMI (kg/m^2^) ± SD ^a^	22 ± 5	24 ± 3	<0.05
Current smoker ^b^			0.446
Yes	11 (17.5)	3 (11.1)	
No	52 (82.5)	24 (88.9)	
Current alcohol consumer ^b^			1.000
Yes	7 (11.1)	3 (11.1)	
No	56 (88.9)	24 (88.9)	
Respiratory comorbidities	11 (17.5)	3 (11.1)	0.758
COPD ^b^	9 (14.3)	3 (11.1)	0.685
Asthma ^c^	2 (3.2)	0 (0)	1.000
Air pollution ^a^			
PM_2.5_ (μg/m^3^)	26.3 ± 2.7	26.3 ± 1.4	0.924
NO_2_ (ppb)	20.7 ± 2.8	19.7 ± 3.3	0.168
SO_2_ (ppb)	3.3 ± 0.7	7.1 ± 0.7	0.455
O_3_ (ppb)	25.6 ± 2.4	26.5 ± 2.5	0.104

^a^ Student’s *t*-test. ^b^ Chi-squared test. ^c^ Fisher’s exact test. BMI = body mass index; COPD = chronic obstructive pulmonary disease; SD = standard deviation; PM_2.5_ = particulate matter with an aerodynamic diameter of <2.5 µm.

**Table 2:** Biomarkers and metals in pleural effusion of 63 patients with pneumonia and 27 patients with congestive heart failure (CHF).

Variable	Pneumonia (*n* = 63)	CHF (*n* = 27)	*p* Value ^a^
Biomarkers			
LDH (U/L)	178 ± 137	131 ± 106	0.140
Total protein (g/dL)	3.1 ± 1.1	2.8 ± 1.3	0.412
CXCL10 (pg/mL)	1195 ± 1066	1294 ± 1061	0.732
IFN-γ (pg/mL)	10 ± 11	7.0 ± 5.9	0.368
CD14 (ng/mL)	15 ± 9	23 ± 14	<0.05
CD62 (ng/mL)	62 ± 24	53 ± 26	0.214
Metals			
Al (ng/mL)	46 ± 35	287 ± 136	<0.01
Cr (ng/mL)	2.7 ± 0.9	1.7 ±0.8	<0.05
Fe (ng/mL)	2116 ± 2924	1815 ± 2540	0.667
Cu (ng/mL)	698 ± 270	626 ± 233	0.250
Zn (ng/mL)	327 ± 157	283 ± 155	0.256
As (ng/mL)	5.3 ± 4.0	3.8 ± 3.0	0.241
Mo (ng/mL)	2.5 ± 1.6	2.9 ± 1.7	0.426
Sn (ng/mL)	2.0 ± 0.7	2.0 ± 1.2	0.816
Pb (ng/mL)	2.4 ± 1.0	2.3 ± 1.3	0.823

^a^ Student’s *t*-test. LDH = lactate dehydrogenase; CXCL10 = C-X-C motif chemokine 10; IFN = interferon; CD = cluster of differentiation.

**Table 3:** Associations of air pollution with normalized biomarkers in the pleural effusion of subjects.

Variable	PM_2.5_ (μg/m^3^)	NO_2_ (ppb)	SO_2_ (ppb)	O_3_ (ppb)
Log_10_ LDH	0.185 (−5.667, 26.624)	−0.342 (−25, 0.044)	0.102 (−0.061, 0.141)	0.169 (−0.090, 0.132)
Log_10_ total protein	−0.065 (−0.036, 0.025)	0.517 (−0.013, 0.047)	−0.054 (−0.089, 0.060)	0.555 (−0.033, 0.127)
Log_10_ CXCL10	−0.033 (−0.066, 0.053)	0.038 (−0.061, 0.066)	−0.025 (−0.163, 0.139)	0.162 (−0.140, 0.193)
Log_10_ IFN−γ	−0.115 (−0.174, 0.088)	−0.732 (−0.157, 0.039)	0.038 (−0.194, 0.241)	−1.047 (−0.514, 0.050)
Log_10_ CD14	−0.086 (−0.068, 0.036)	−0.397 (−0.055, 0.022)	0.104 (−0.060, 0.128)	−0.582 (−0.170, 0.042)
Log_10_ CD62	−0.213 (−0.071, 0.015)	−0.979 (−0.085, −0.004) *	0.210 (−0.026, 0.169)	−0.739 (−0.192, 0.021)

Note: Values are beta coefficients and 95% confidence intervals after adjusting for age, sex, body mass index, smoking, and disease category (pneumonia or congestive heart failure) in the models. * *p* < 0.05. PM_2.5_ = particulate matter with an aerodynamic diameter of <2.5 µm; LDH = lactate dehydrogenase; CXCL10 = C-X-C motif chemokine 10; IFN = interferon; CD = cluster of differentiation.

**Table 4:** Associations of air pollution with normalized levels of metals in the pleural effusion of subjects.

Variable	PM_2.5_ (μg/m^3^)	NO_2_ (ppb)	SO_2_ (ppb)	O_3_ (ppb)
Log_10_ Al	−0.085 (−0.084, 0.044)	−0.419 (−0.104, 0.032)	−0.043 (−0.180, 0.120)	−0.290 (−0.242, 0.114)
Log_10_ Cr	−0.585 (−0.156, 0.047)	−0.371 (−0.083, 0.065)	0.207 (−0.151, 0.252)	−0.251 (−0.205, 0.173)
Log_10_ Fe	−0.199 (−0.098, 0.017)	−0.724 (−0.112, 0.012)	−0.156 (−0.235, 0.055)	−0.482 (−0.253, 0.078)
Log_10_ Cu	0.045 (−0.025, 0.033)	0.584 (−0.010, 0.045)	−0.015 (−0.066, 0.059)	0.701 (−0.018, 0.129)
Log_10_ Zn	−0.143 (−0.047, 0.016)	0.255 (−0.025, 0.044)	−0.275 (−0.164, −0.006) *	0.493 (−0.044, 0.138)
Log_10_ As	−0.178 (−0.094, 0.027)	0.775 (−0.020, 0.099)	0.184 (−0.052, 0.217)	0.893 (−0.038, 0.274)
Log_10_ Mo	−0.269 (−0.127, 0.018)	−1.590 (−0.138, −0.020) *	−0.263 (−0.236, 0.023)	−1.599 (−0.372, −0.053) *
Log_10_ Sn	−0.655 (−0.182, 0.075)	−0.994 (−0.186, 0.125)	0.073 (−0.285, 0.326)	−0.154 (−0.324, 0.300)
Log_10_ Pb	0.173 (−0.140, 0.194)	−0.505 (−0.129, 0.096)	0.140 (−0.269, 0.277)	−0.419 (−0.376, 0.260)

Note: Values are beta coefficients and 95% confidence intervals after adjusting for age, sex, body mass index, smoking, and disease category (pneumonia or CHF) in the models. * *p* < 0.05. PM_2.5_ = particulate matter with an aerodynamic diameter of <2.5 µm.
